# Optimal hemoglobin threshold for blood transfusions in sepsis and septic shock: a retrospective analysis

**DOI:** 10.1007/s11739-025-03889-4

**Published:** 2025-02-20

**Authors:** Chairat Permpikul, Jakpanee Tanksinmankhong, Surat Tongyoo, Thummaporn Naorungroj, Tanuwong Viarasilpa, Khemajira Karaketklang

**Affiliations:** 1 Division of Critical Care, Department of Internal Medicine, Faculty of Medicine, Siriraj Hospital, Mahidol University, 2 Prannok Road, Bangkok Noi, Bangkok, 10700 Thailand; 2 Department of Internal Medicine, Faculty of Medicine, Siriraj Hospital, Mahidol University, Bangkok, Thailand

**Keywords:** Hemoglobin level, Liberal blood transfusion, RBC transfusion, Restrictive blood transfusion, Sepsis, Septic shock

## Abstract

Transfusions of red blood cells (RBCs) are crucial for improving tissue oxygenation in anemic patients with sepsis. Nevertheless, the debate continues over the ideal hemoglobin level for transfusions. This study aimed to assess the impact of different hemoglobin levels on the outcomes of patients with sepsis who received transfusions. This retrospective analysis included adult patients with sepsis treated in the general medical ward and intensive care unit at a University affiliate hospital. Patients needing RBC transfusions were included. The primary outcome was the 28-day mortality rate. From March 2018 to January 2022, 806 patients were studied. Of these, 480 (59.6%) were transfused at hemoglobin levels of 7–9 g/dL (“liberal group”), while 326 (40.4%) received RBC transfusions when their hemoglobin was < 7 g/dL (“restrictive group”). Mean hemoglobin levels at transfusion were 8.1 ± 0.8 g/dL and 6.3 ± 0.8 g/dL for each group, respectively(*P* < 0.001). On day 28, the liberal group had a mortality rate of 51.2% (246 patients), compared to 59.2% (193 patients) in the restrictive group (Odds ratio [OR] 0.88, 95% confidence interval [CI] 0.79–0.98, *P* = 0.031). Adjusted comparisons showed 46.8% mortality in the liberal group (141/301patients) versus 59.3% in the restrictive group (178/300patients) at 28 days (OR 0.78, 95% CI 0.66–0.92, *P* = 0.002). Multivariate analysis revealed transfusion at hemoglobin 7–9 g/dL as an independent variable linked to lower 28-day mortality (OR 0.70, 95% CI 0.49–0.99, *P* = 0.042). Other factors correlated with 28-day mortality were platelet counts ≤ 150 × 10^3^/µL, albumin ≤ 2.5 g/dL, shock, mechanical ventilation, and renal replacement therapy. This retrospective study suggests that RBC transfusion at hemoglobin levels of 7–9 g/dL associates with lower 28-day mortality in sepsis patients compared to transfusion at hemoglobin levels below 7 g/dL.

**Clinical trial registration**

The study was registered with the Thai Clinical Trials Registry (identification number TCTR20231003003). (https://www.thaiclinicaltrials.org/show/TCTR20231003003).

**Supplementary Information:**

The online version contains supplementary material available at 10.1007/s11739-025-03889-4.

## Background

Sepsis and septic shock represent a critical range of organ failure caused by infections, often leading to high mortality rates even with the best possible care [1–4]. As outlined in the Survival Sepsis campaign [5, 6], effective management includes providing hemodynamic resuscitation [7], controlling the infection, and offering optimal support for affected organs. A vital component of this organ support is the transfusion of red blood cells (RBCs), which aims to restore and improve tissue blood flow and oxygenation. However, blood transfusions carry potential risks and can sometimes be detrimental [8]. Complications such as transfusion-associated circulatory overload, transfusion-related acute lung injury [9], allergic reactions, and the transmission of diseases through transfusions have been documented. While guidelines exist [10, 11], they leave room for debate, particularly regarding the benefits of transfusion on microcirculation and the appropriate hemoglobin level for initiating transfusion.

The microcirculatory effects of RBC transfusion in sepsis have been extensively studied [12]. Research has shown that RBC transfusion can increase mixed/central venous oxygen saturation, yet capillary oxygen tension remains low [13]. In addition, direct sublingual video microscopy demonstrated that RBC transfusion did not significantly alter microcirculatory flow, although some positive effects were noted in patients with altered capillary perfusion at baseline [14]. It was also found that leukocyte-depleted RBCs enhanced the microvascular flow index and blood flow velocity but did not affect other microcirculatory parameters [15].

Extensive studies have revealed consistent findings regarding the optimal hemoglobin level for transfusion. Hébert and the Transfusion Requirements in Critical Care trial group conducted a randomized controlled trial on transfusion triggers and targets in critically ill patients [16]. They compared two strategies. The “restrictive strategy” initiated transfusions when hemoglobin levels fell below 7.0 g/dL, maintaining levels between 7 and 9 g/dL. Conversely, the “liberal strategy” set the trigger and target levels at 9 and 10–12 g/dL, respectively. They found that 30-day mortality rates were similar between the two strategies, although younger patients with milder disease severity showed lower mortality under the restrictive strategy. Following this, Holst and the Transfusion Requirements in Septic Shock trial group reported similar findings in a multicenter trial involving patients with septic shock [17]. The trial randomized patients to receive 1 unit of leukoreduced red cells at a lower threshold of 7 g/dL or a higher threshold of 9 g/dL during their intensive care unit stay and the primary outcome was death by 90 days after randomization. The results showed that the mortality at 90 days and rates of ischemic events were similar among groups. These findings closely mirrored those of the Transfusion Requirements in Critical Care trial, reinforcing the guidelines based on these findings [5, 6, 18].

However, contrasting results have emerged from other studies. For instance, the Transfusion Requirements in Critically Ill Oncologic Patients study, which focused on oncologic patients with septic shock, indicated that a liberal transfusion strategy led to better survival rates [19]. In addition, data from our institution revealed that the mortality rate in patients with septic shock and lower hemoglobin levels at Siriraj Hospital was 43.9%. This level was 1.5 times higher than the rate observed in the higher hemoglobin group (34.3%) [20].

The optimal hemoglobin level for initiating transfusions in patients with septic shock remains a topic of debate. To address this, we carried out a retrospective study focusing on patient outcomes following blood transfusions at varying hemoglobin levels. The primary measure of this study was the mortality rate at 28 days. Secondary outcomes included the duration of hospital stay and complications associated with sepsis or septic shock.

## Methods

### Study design and ethical considerations

This retrospective cohort study focused on adult patients with sepsis or septic shock. These patients were admitted to the general ward or the medical intensive care unit at Siriraj Hospital, Mahidol University, in Bangkok, Thailand. The study protocol was authorized by the Institutional Review Board of Siriraj Hospital (approval number Si-556/2022). Due to the retrospective nature of the study, the requirement for informed consent was waived. To ensure patient privacy and confidentiality, all data collection was conducted anonymously, ensuring no inclusion of names or personal identifiers.

### Participants

We identified patients diagnosed with sepsis and septic shock based on the corresponding International Classification of Diseases, Tenth Revision codes. The inclusion criteria were adult patients (18 years or older) admitted from March 1, 2018, to January 31, 2022. These patients met the diagnostic criteria for sepsis or septic shock outlined in the Surviving Sepsis Campaign: International Guidelines for Management of Severe Sepsis and Septic Shock 2016 [5]. In addition, they received packed RBC (PRBC) transfusions within 3 days following their sepsis or septic shock diagnosis. “Septic shock” was defined as either hypotension with a mean arterial blood pressure below 65 mm Hg or a condition requiring vasopressor support to maintain a mean ≥ 65 mm Hg. Both conditions also needed to be accompanied by a confirmed infection and signs of systemic inflammation.

Patients were excluded if they had any of the following:

- significant bleeding (characterized by overt bleeding leading to a systolic blood pressure drop of over 20 mmHg, a heart rate increase ≥ 20 beats per minute, or a hemoglobin decrease of more than 2 g/dL);
- acute coronary syndrome;
- explicit gastrointestinal bleeding;
- immediate postoperative status.

All participants underwent resuscitation according to a septic shock protocol. It involved fluid resuscitation, vasopressor therapy if necessary, prompt initiation of antimicrobial treatment within 1 h, proper source control, and organ support. The decision to perform an RBC transfusion was at the attending physician’s discretion. Patients receiving RBC transfusions who had hemoglobin levels between 7 and 9 g/dL were categorized as the “liberal group.” Patients with hemoglobin levels below 7 g/dL were placed in the “restrictive group.”

### Data collection and outcomes

The patients’ electronic medical records were thoroughly reviewed to gather relevant data. Information regarding baseline characteristics, clinical data, and outcomes was extracted. The baseline characteristics encompassed age, sex, body mass index, and comorbidities. The clinical data included the site of infection, baseline vital signs, and severity score. In addition, all relevant laboratory investigations were obtained from the hospital’s online database.

The study’s primary outcome was the 28-day mortality rate among the patients. The secondary outcomes focused on the length of hospital stay and the complications arising from sepsis or septic shock. These complications specifically included acute pulmonary edema, acute kidney injury, acute respiratory distress syndrome, cardiac arrest, and bowel ischemia.

### Statistical analysis

In this study, continuous data are represented either as the mean ± standard deviation or the median with the interquartile range. The independent *t* test was utilized to analyze continuous variables following a normal distribution. For continuous variables that did not distribute normally, the Mann–Whitney *U* test was employed. Categorical data are presented as percentages. To compare categorical variables, either the chi-square test or Fischer’s exact test was employed.

To enhance comparability and minimize selection bias, we employed a one-to-one ratio to match patients in the liberal and restrictive groups based on baseline characteristics. Propensity score matching was used to achieve balance for potential confounding factors, including: age, sex, body mass indexes, baseline severity scores, underlying conditions, and baseline hemoglobin levels, using a nearest neighbor matching model with the R software package “matchIt” was used. A 1:1 ratio to match the liberal group to the restrictive group with standard mean difference (SMD) less than 0.05 was considered balanced in these covariates, on the logit of the estimated propensity score [21].

The primary outcome, 28-day mortality, was evaluated using the chi-square test. In evaluating the 28-day mortality, we calculated the time from the diagnosis of sepsis or septic shock to the date of death. Survival distributions in the liberal and restrictive groups were estimated by plotting Kaplan–Meier curves. The hazard ratio of 28-day mortality was calculated using the Cox proportional hazards model. This outcome assessment was conducted for both the overall group and the matched comparisons. Our analysis considered a *P* value of less than 0.05 statistically significant.

To ascertain predictive factors related to 28-day mortality, we categorized patients into two groups: survivors and nonsurvivors. A comparative analysis was conducted between these two groups within each classification. We employed receiver operating characteristic analysis to establish cutoff values for continuous variables that exhibited significant differences between the survivors and nonsurvivors. Youden’s index was utilized to determine the optimal cutoff for each variable, along with its corresponding sensitivity and specificity [22].

Following the receiver operating characteristic analysis, variables were reclassified according to the identified cutoff values. We then conducted univariate analyses on these reclassified variables, expressing risk as an unadjusted Odds ratio (OR) with a 95% confidence interval (CI). Predictive factors with a *P* value of 0.1 or less, along with other relevant factors, were included in binary logistic regression analyses. The outcomes of the multivariate analysis are presented as adjusted ORs with 95% CIs and *P* values. Factors identified as independently predictive of 28-day mortality were those with a *P* value of 0.05 or less.

All statistical procedures were performed using IBM SPSS Statistics, version 26 (IBM Corp, Armonk, NY, USA).

## Results

**Fig. 1:**
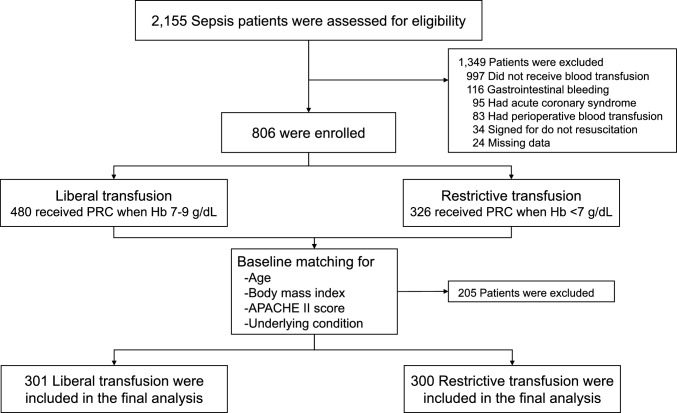
Enrollment and exclusion flow diagram of patient study groups

We reviewed the charts of 2155 patients with sepsis, ultimately enrolling 806 of them. Of these, 480 patients who received PRBC transfusions at hemoglobin levels ranging from 7 to 9 g/dL were categorized as the liberal group. The remaining 326 patients received transfusions at hemoglobin levels below 7 g/dL and were placed in the restrictive group (Fig. 1).

Table 1 details the demographic and clinical characteristics of the patients in the two groups. The liberal group exhibited a significantly higher mean age (67.4 ± 16.5 vs 63.8 ± 16.1 years, *P* = 0.003), body mass index (22.6 ± 5.4 vs 21.5 ± 5.9 kg/m^2^, *P* = 0.014), and baseline hemoglobin level (9.9 ± 2.2 vs 8.2 ± 2.4 g/dL, *P* = 0.001). The incidence of malignancy as an underlying condition was lower in the liberal group. Pneumonia was the most common type of infection, followed by septicemia, intra-abdominal infections, urinary tract infections, and soft tissue infections.

**Table 1:** Baseline characteristics of patients and treatment modalities administered during sepsis shock resuscitation

Baseline parameters	Overall				Baseline matched			
	All (*n* = 806)	Hb 7–9 (*n* = 480)	Hb < 7 (*n* = 326)	*P*	All (*n* = 601)	Hb 7–9 (*n* = 301)	Hb < 7 (*n* = 300)	*P*
Age, mean ± SD, year	65.9 ± 16.4	67.4 ± 16.5	63.8 ± 16.1	0.003	64.8 ± 16.5	65.3 ± 17.1	64.3 ± 16.1	0.439
BMI, mean ± SD, kg/m^2^	22.1 ± 5.6	22.6 ± 5.4	21.5 ± 5.9	0.014	21.6 ± 5.3	21.7 ± 4.6	21.5 ± 5.9	0.789
APACHE II,* mean ± SD	20.1 ± 7.4	19.2 ± 6.9	21.4 ± 7.8	0.062	20.3 ± 7.9	19.3 ± 7.7	21.2 ± 8.0	0.179
Sex, male, no (%)	434 (53.8)	268 (55.8)	166 (50.9)	0.170	335 (55.7)	178 (59.1)	157 (52.3)	0.110
Underlying, no (%)								
Hypertension	416 (51.6)	258 (53.8)	158 (48.5)	0.141	296 (49.3)	145 (48.2)	151 (50.3)	0.596
Malignancy	297 (36.8)	171 (35.6)	126 (38.7)	0.020	239 (39.8)	123 (40.9)	116 (38.7)	0.582
Diabetes mellitus	252 (31.3)	157 (32.7)	95 (29.1)	0.284	182 (30.3)	92 (30.6)	90 (30.0)	0.880
Chronic kidney disease	190 (23.6)	114 (23.8)	76 (23.3)	0.886	144 (24.0)	72 (23.9)	72 (24.0)	0.982
Coronary artery disease	107 (13.3)	71 (14.8)	36 (11.0)	0.124	77 (12.8)	43 (14.3)	34 (11.3)	0.279
Cerebrovascular disease	100 (12.4)	61 (12.7)	39 (12.0)	0.753	72 (12.0)	35 (11.6)	37 (12.3)	0.790
Cirrhosis	78 (9.7)	53 (11.0)	25 (7.7)	0.112	59 (9.8)	35 (11.6)	24 (8.0)	0.135
Chronic lung disease	45 (5.6)	30 (6.3)	15 (4.6)	0.314	28 (4.7)	13 (4.3)	15 (5.0)	0.699
HIV	23 (2.9)	11 (2.3)	12 (3.7)	0.245	21 (3.5)	9 (3.0)	12 (4.0)	0.500
Infection type, no (%)				0.455				0.289
Pneumonia	305 (37.8)	183 (38.1)	122 (37.4)		228 (37.9)	111 (36.9)	117 (39.0)	
Septicemia	158 (19.6)	92 (19.2)	66 (20.2)		122 (20.3)	62 (20.6)	60 (20.0)	
Intra-abdominal infection	110 (13.6)	74 (15.4)	36 (11.1)		84 (14.0)	49 (16.3)	35 (11.7)	
Urinary tract infection	86 (10.7)	44 (9.2)	42 (12.9)		67 (11.1)	28 (9.3)	39 (13.0)	
Soft tissue infection	26 (3.2)	17 (3.5)	9 (2.8)		20 (3.3)	12 (4.0)	8 (2.7)	
CRBSI	9 (1.1)	4 (0.8)	5 (1.5)		3 (0.5)	0 (0.0)	3 (1.0)	
CNS infection	4 (0.5)	2 (0.4)	2 (0.6)		3 (0.5)	1 (0.3)	2 (0.7)	
Unknown	108 (13.4)	64 (13.3)	44 (13.5)		74 (12.3)	38 (12.6)	36 (12.0)	
Vital signs, mean ± SD								
Temperature	37.2 ± 1.4	37.3 ± 0.9	37.2 ± 1.8	0.703	37.3 ± 1.0	37.3 ± 1.0	37.3 ± 0.9	0.746
Heart rate	100.9 ± 43.9	101.0 ± 54.1	100.7 ± 21.6	0.922	99.8 ± 22.6	99.0 ± 23.5	100.5 ± 21.7	0.410
Mean blood pressure	83.4 ± 19.3	84.2 ± 19.2	82.1 ± 19.3	0.129	82.5 ± 19.0	82.3 ± 18.3	82.8 ± 19.6	0.745
Respiratory rate	26.2 ± 8.2	26.6 ± 9.0	25.7 ± 6.9	0.158	25.9 ± 8.1	26.0 ± 9.0	25.6 ± 7.0	0.697
Oxygen saturation	94.6 ± 7.3	94.3 ± 7.6	95.0 ± 6.8	0.208	94.8 ± 7.2	94.7 ± 7.4	94.8 ± 7.0	0.857
Laboratory findings								
Hemoglobin, g/dL	9.2 ± 2.4	9.9 ± 2.2	8.2 ± 2.4	0.001	8.6 ± 1.8	8.7 ± 1.4	8.6 ± 2.1	0.427
WBC, × 10^3^/µL	13.9 ± 18.8	12.9 ± 14.0	15.3 ± 24.0	0.105	14.0 ± 18.0	13.4 ± 15.2	14.6 ± 21.8	0.426
Platelet, × 10^3^/µL	197.5 ± 146.4	198.0 ± 136.9	196.8 ± 159.5	0.905	196.9 ± 154.2	198.4 ± 153.1	195.5 ± 155.5	0.816
Creatinine, mg/dL	2.1 ± 2.3	2.0 ± 2.1	2.3 ± 2.5	0.158	2.0 ± 2.0	1.9 ± 1.9	2.1 ± 2.1	0.133
Serum albumin, g/dL	2.8 ± 0.8	2.9 ± 0.8	2.8 ± 0.8	0.170	2.8 ± 0.8	2.8 ± 0.8	2.8 ± 0.8	0.903
Serum lactate, mmol/L	4.7 ± 4.9	4.8 ± 5.0	4.5 ± 4.8	0.510	4.5 ± 4.7	4.4 ± 4.5	4.5 ± 4.9	0.815
Organ support								
Mechanical ventilator	601 (74.6)	357 (74.4)	244 (74.8)	0.880	435 (72.4)	208 (69.1)	227 (75.7)	0.088
Vasopressor	513 (63.6)	312 (65.0)	201 (61.7)	0.333	377 (62.7)	189 (62.8)	188(62.7)	0.975
RRT	212 (26.4)	127 (26.5)	85 (26.3)	0.950	146 (24.4)	66 (22.0)	80 (26.8)	0.199
Surgical drainage	78 (9.7)	60 (12.5)	18 (5.5)	0.001	57 (9.5)	40 (13.3)	17 (5.7)	0.001
Blood component transfusion								
PRBC units	1 (1–2)	1 (1–2)	2 (1–3)	< 0.001	2 (1–3)	1 (1–2)	2 (1–3)	< 0.001
Hb at transfusion, g/dL	7.3 ± 1.2	8.1 ± 0.8	6.3 ± 0.8	< 0.001	7.1 ± 1.0	7.9 ± 0.7	6.4 ± 0.7	< 0.001
Platelet	282 (35.0)	167 (34.8)	115 (35.3)	0.887	203 (33.8)	99 (32.9)	104 (34.7)	0.645
FFP	301 (37.3)	176 (36.7)	125 (38.3)	0.629	217 (36.1)	101 (33.6)	116 (38.7)	0.192
Cryoprecipitate	91 (11.3)	55 (11.5)	36 (11.0)	0.855	57 (9.5)	25 (8.3)	32 (10.7)	0.323

*APACHE* acute physiology and chronic health evaluation, *BMI* body mass index, *CNS* central nervous system, *CRBSI* catheter-related blood stream infection, *HIV* human immunodeficiency virus, *SD* standard deviation, *WBC* white blood cell count, FFP fresh frozen plasma, *Hb* hemoglobin level, *PRBC* packed red blood cell, *RRT* renal replacement therapy

^*^Includes APACHE II scores, which are severity assessment metrics ranging from 0 to 71, with higher scores indicating more severe disease

^*^Data are represented as number (percentage), median (interquartile range), or mean ± standard deviation, as applicable

After performing baseline matching, the final analysis included 601 patients: 301 received liberal RBC transfusions, and the remaining 300 were administered restrictive RBC transfusions. No significant differences in baseline characteristics were observed between the two groups.

### Treatment variables

Table 1 provides an overview of all treatment variables. Most patients needed mechanical ventilator support, with no significant difference observed between the liberal and restrictive groups (74.4% vs 74.8%, *P* = 0.880). Septic shock necessitating vasopressors was reported in 65.0% of the patients in the liberal group and 61.7% in the restrictive group (*P* = 0.333). Renal replacement therapy was performed in a similar proportion in both groups (26.5% in the liberal group vs 26.3% in the restrictive group, *P* = 0.950). However, surgical drainage was performed significantly more frequently in the liberal group than in the restrictive group (12.5% vs 5.5%, *P* = 0.001).

The triggering hemoglobin level was significantly higher in the liberal group than in the restrictive group (8.1 ± 0.8 vs 6.3 ± 0.8 g/dL, *P* < 0.001). Furthermore, patients in the liberal group received a lower median (interquartile range) number of PRBC transfusions than those in the restrictive group (1 [1, 2] vs 2 [1–3], *P* < 0.001). There were no significant differences between the two groups regarding transfusion of other blood components (platelet concentrate, fresh frozen plasma, and cryoprecipitate).

### Treatment outcomes

**Fig. 2:**
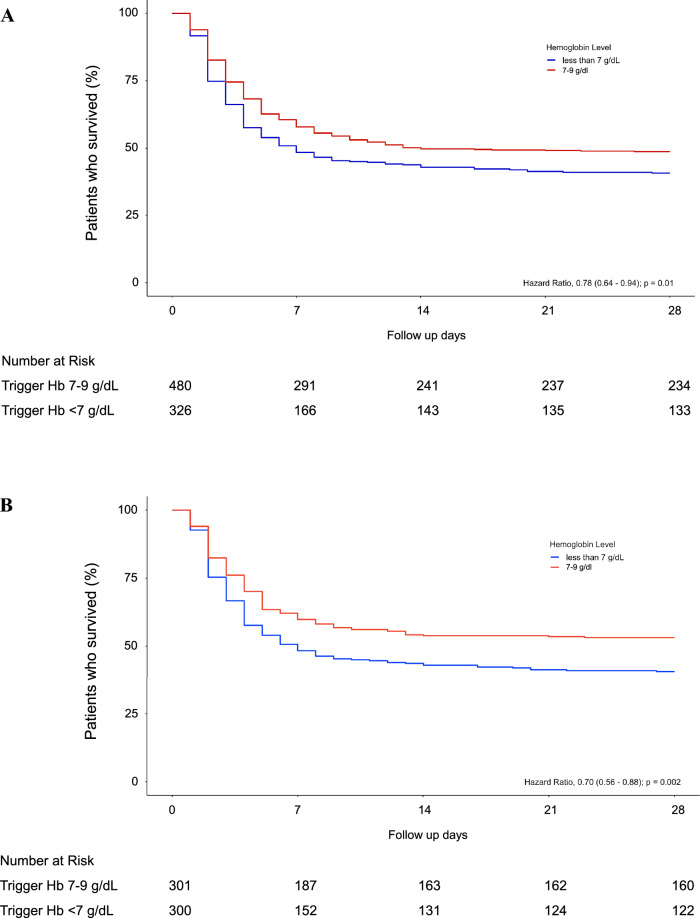
Kaplan–Meier curve analysis for 28-day survival probability. **A** Overall population analysis. The hazard ratio for mortality in the liberal group (7–9 g/dL) compared to the restrictive group (< 7 g/dL) was 0.78 (95% confidence interval 0.64–0.94, *P* = 0.01). **B** Baseline matched population analysis. The hazard ratio for mortality in the liberal group (7–9 g/dL) compared to the restrictive group (< 7 g/dL) was 0.70 (95% confidence interval 0.56–0.88, *P* = 0.002)

The treatment outcomes of the patients are presented in Table 2. In the analysis of the overall population, the 28-day mortality rate was 51.2% in the liberal group and 59.2% in the restrictive group. This difference translates to an OR of 0.88, with a 95% CI of 0.79–0.98 and a *P* value of 0.031. In the baseline-matched comparison, the 28-day mortality rate was 46.8% in the liberal group and 59.3% in the restrictive group (OR 0.78, 95% CI 0.66–0.92, *P* = 0.002). Figure 2 illustrates the Kaplan–Meier curves for 28-day mortality.

**Table 2:** Clinical outcomes of patients

Outcome variables*	Overall					Baseline matched				
	All (*n* = 806)	Hb 7–9 (*n* = 480)	Hb < 7 (*n* = 326)	OR (95% CI)	*P*	All (*n* = 601)	Hb 7–9 (*n* = 301)	Hb < 7 (*n* = 300)	OR (95% CI)	*P*
28-day mortality	439 (54.5)	246 (51.2)	193 (59.2)	0.88 (0.79–0.98)	0.031	319 (53.1)	141 (46.8)	178 (59.3)	0.78 (0.66–0.92)	0.002
Hospital LOS	23.5 ± 19.6	23.9 ± 19.2	22.9 ± 20.1		0.49	22.5 ± 18.7	22.4 ± 17.9	22.7 ± 19.6		0.83
Complications										
- AKI	469 (58.2)	280 (58.3)	189 (58.0)	1.01 (0.89–1.12)	0.919	339 (56.4)	166 (54.8)	174 (57.0)	0.93 (0.80–1.10)	0.431
- Pulmonary edema	131 (16.3)	84 (17.5)	47 (14.4)	1.09 (0.94–1.27)	0.244	98 (16.3)	53 (17.6)	45 (15.0)	1.10 (0.89–1.35)	0.387
- ARDS	99 (12.3)	58 (12.1)	41 (12.6)	0.98 (0.82–1.18)	0.834	74 (12.3)	35 (11.6)	39 (13.0)	0.93 (0.72–1.20)	0.609
- Cardiac arrest	71 (8.8)	41 (8.5)	30 (9.2)	0.97 (0.79–1.19)	0.745	51 (8.5)	23 (7.6)	28 (9.3)	0.89 (0.65–1.22)	0.458
- Bowel ischemia	11 (1.4)	6 (1.2)	5 (1.5)	0.92 (0.53–1.56)	0.764	7 (1.2)	2 (0.7)	5 (1.7)	0.57 (0.18–1.82)	0.252

*AKI* acute kidney injury, *ARDS* acute respiratory distress syndrome, *Hb* hemoglobin, *LOS* length of stay

^*^Data are presented as either number (percentage) or mean ± standard deviation, where relevant

For the overall population, the hazard ratio for death in the liberal group compared to the restrictive group was 0.78 (95% CI 0.64–0.94, *P* = 0.01). In the baseline-matched population, the hazard ratio for the same comparison was 0.70 (95% CI 0.56–0.88, *P* = 0.002).

The incidence of complications did not significantly differ between the two groups. Overall, acute kidney injury was the most frequent complication (58.2%), followed by acute pulmonary edema (16.3%), acute respiratory distress syndrome (12.3%), cardiac arrest (8.8%), and acute bowel ischemia (1.4%).

### Multivariate and subgroup analyses

A multivariate analysis was conducted to identify the predictive factors independently associated with 28-day mortality. The analysis included all 12 clinical parameters that showed a potentially significant difference (*P* value less than 0.1) between survivors and nonsurvivors in the univariate analysis (Table 3). Out of the 12 parameters, six remained significant in the final multivariate model.

**Table 3:** Multivariate analysis: identifying predictive factors for 28-day mortality

Clinical variables	Univariate analysis		Multivariate analysis	
	Odds ratio (95% CI)	*P*	Odds ratio (95% CI)	*P*
Age > 80 years	1.19 (0.98–1.45)	0.069		
Heart failure	1.75 (0.82–3.72)	0.089		
HIV	1.77 (0.89–3.03)	0.057		
Hb baseline ≤ 9 g/dl	1.14 (0.98–1.32)	0.096		
Platelet ≤ 150 × 10^3^/µL	1.28 (1.10–1.50)	0.002	1.70 (1.21–2.39)	0.002
Lactate > 2	1.41 (1.21–1.66)	< 0.001		
Albumin ≤ 2.5 g/dL	1.26 (1.05–1.51)	0.009	1.76 (1.23–2.50)	0.002
Shock	1.46 (1.26–1.69)	< 0.001	2.06 (1.44–2.95)	< 0.001
Mechanical ventilation	1.98 (1.73–2.27)	< 0.001	4.46 (2.98–6.67)	< 0.001
RRT	1.49 (1.21–1.83)	< 0.001	1.78 (1.19–2.67)	0.005
Drainage	0.79 (0.64–0.97)	0.042		
Hb trigger 7–9 g/dL	0.86 (0.76–0.98)	0.026	0.70 (0.49–0.99)	0.042

*Hb* hemoglobin, *HIV* human immunodeficiency virus, *RRT* renal replacement therapy

The only parameter identified as a protective factor against 28-day mortality was triggering RBC transfusion at hemoglobin levels between 7 and 9 g/dL (OR 0.70, 95% CI 0.49–0.99, *P* = 0.042). Independent predictive factors associated with increased 28-day mortality were baseline platelets ≤ 150 × 10^3^/µL (OR 1.70, 95% CI 1.21–2.39, *P* = 0.002), baseline serum albumin ≤ 2.5 g/dL (OR 1.76, 95% CI 1.23–2.50, *P* = 0.002), the need for vasopressors in septic shock (OR 2.06, 95% CI 1.44–2.95, *P* < 0.001), receiving mechanical ventilator support (OR 4.46, 95% CI 2.98–6.67, *P* < 0.001), and receiving renal replacement therapy (OR 1.78, 95% CI 1.19–2.67, *P* = 0.005).

**Fig. 3:**
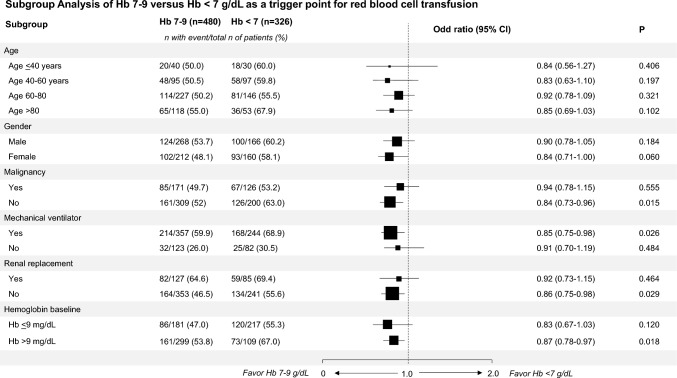
Comparative subgroup analysis based on hemoglobin levels (7–9 g/dL vs < 7 g/dL) as the trigger for RBC transfusions

A subgroup analysis was also performed (Fig. 3). Four clinical parameters were associated with survival benefit when RBC transfusion was triggered at a hemoglobin level of 7–9 g/dL. Patients who met one or more of the following criteria were more likely to survive:

- needed mechanical ventilation support (OR 0.85, 95% CI 0.75–0.98, *P* = 0.026);
- did not have malignancy (OR 0.84, 95% CI 0.73–0.96, *P* = 0.015);
- did not require renal replacement therapy (OR 0.86, 95% CI 0.75–0.98, *P* = 0.029);
- had baseline hemoglobin levels above 9 g/dL (OR 0.87, 95% CI 0.78–0.97, *P* = 0.018).

## Discussion

Our study focused on patients with sepsis and septic shock who had anemia without clinically significant bleeding. We observed that patients who received RBC transfusions at high hemoglobin levels (≥ 7 g/dL) had a lower 28-day mortality rate than those transfused at lower hemoglobin levels. This finding was consistent in both the overall and the baseline-matched models. However, we did not observe any significant differences in the lengths of hospital stay or the occurrence of complications.

In addition to transfusion at a hemoglobin level of less than 7 g/dL, our multivariate analysis identified several other factors associated with a higher 28-day mortality rate. These factors included being in a state of shock, having a platelet count of ≤ 150 000/cu.mm, having serum albumin levels ≤ 2.5 g/dL, and needing renal replacement therapy or mechanical ventilation.

The main finding of our study showed some differences from the results of the Transfusion Requirements in Critical Care trial and the Transfusion Requirements in Septic Shock trial [16, 17]. These major trials were designed to compare outcomes between patients receiving RBC transfusions at hemoglobin levels < 7 g/dL (the restrictive group) and those administered transfusions at the higher threshold of 9 g/dL (the liberal group). In our study, we compared patients given transfusions at hemoglobin levels below 7 g/dL to those receiving transfusions at hemoglobin levels between 7 and 9 g/dL. Our findings indicated that the group with higher hemoglobin levels (7–9 g/dL) had lower mortality than the group with levels below 7 g/dL. This supports the notion that maintaining hemoglobin levels at not less than 7 g/dL, or preferably within the 7–9 g/dL range, may be more beneficial for patients with sepsis.

Furthermore, the Transfusion Requirements in Critical Care trial and the Transfusion Requirements in Septic Shock trial were designed with two thresholds (7 and 9 g/dL) to initiate blood transfusion. These thresholds might not always have aligned with patients’ immediate clinical indications for RBC transfusion. In our study, transfusion decisions were based on the attending physicians’ judgments, primarily influenced by patients’ clinical and hemodynamic statuses. However, due to the differences in the nature of randomized controlled studies and retrospective studies, caution is needed when comparing results across different studies.

Our subgroup analysis revealed that patients with baseline hemoglobin levels above 9 g/dL had better survival outcomes when transfusions were initiated at these higher hemoglobin levels. This suggests that early transfusion may be particularly beneficial for patients presenting with higher hemoglobin levels. However, this observation should be interpreted with caution and does not imply that transfusions should be initiated solely based on hemoglobin levels above 9 g/dL. Further research is needed to confirm the potential benefits of early transfusion in patients with initially high hemoglobin levels.

Regarding oncologic patients, our subgroup analysis did not support the findings of the Transfusion Requirements in Critically Ill Oncologic Patients study [19], which reported lower mortality in patients receiving liberal blood transfusions. This discrepancy could be due to an insufficient number of patients in our study to show a significant mortality benefit. In addition, our cohort included a substantial number of patients with hematologic malignancies. A prior retrospective study suggested that RBC transfusion during the early stages of resuscitation for severe sepsis and septic shock in patients with hematological malignancies might be linked to increased mortality risk [23].

Turning to the acute complications of RBC transfusions, a meta-analysis encompassing 12 cohort studies indicated that while RBC transfusion was not associated with increased mortality, it was related to high incidences of acute lung injury and acute kidney injury [24]. However, our current study found no significant difference in the incidence of acute respiratory distress syndrome, acute pulmonary edema, or acute kidney injury between the liberal and restrictive groups.

The strengths of our study lie in including a relatively large number of participants and comprehensively collecting data related to baseline clinical characteristics and laboratory investigations. We also analyzed the 28-day mortality outcomes using both overall and baseline-matched models. The findings from our multivariate analysis, which identified the trigger for RBC transfusion at a hemoglobin level of 7–9 g/dL as an independent protective factor against 28-day mortality, corroborated the comparative findings.

However, our study had limitations due to its retrospective nature. These included potential biases and missing data in medical records. In some cases, crucial information was not fully documented, such as the timing of the septic shock diagnosis or the initiation of RBC transfusion, instances of multiple transfusion episodes, and the average hemoglobin level throughout admission. To more definitively address these questions, well-designed prospective randomized controlled trials with adequate power are necessary. Nevertheless, our study offers preliminary insights and identifies potential areas for further investigation. We hope that our findings will lay the groundwork for future randomized controlled trials to elucidate the role of hemoglobin levels in the management of sepsis.

## Conclusions

This study showed that RBC transfusions at hemoglobin levels of 7–9 g/dL reduced mortality in patients with septic shock compared to transfusions below 7 g/dL. However, no significant differences were observed in the hospital lengths of stay or incidences of septic shock complications between the two groups. While these findings partially align with previous randomized controlled trials and guidelines recommending RBC transfusion, they also present inconsistencies with evidence from earlier studies. Consequently, further research is necessary to confirm these results and establish the optimal transfusion threshold in this patient population.

## Supplementary Information

Below is the link to the electronic supplementary material.

Supplementary file1 (DOCX 32 KB)

## Data Availability

For data-sharing requests, researchers can reach the corresponding author at surat.ton@mahidol.ac.th. After a proposed analysis protocol gains approval, anonymised participant data will be accessible 3 months post-publication.

## Abbreviations

RBC: Red blood cell
PRBC: Packed red blood cell
